# Citral-Enriched Fraction of Lemon Essential Oil Mitigates LPS-Induced Hepatocyte Injuries

**DOI:** 10.3390/biology12121535

**Published:** 2023-12-17

**Authors:** Roberta Gasparro, Marzia Pucci, Elisa Costanzo, Ornella Urzì, Vincenza Tinnirello, Marta Moschetti, Alice Conigliaro, Stefania Raimondo, Valeria Corleone, Simona Fontana, Riccardo Alessandro

**Affiliations:** 1 Department of Biomedicine, Neurosciences, and Advanced Diagnostics (Bi.N.D), Section of Biology, Via Divisi 83, University of Palermo, 90133 Palermo, Italy; roberta.gasparro@unipa.it (R.G.); marzia.pucci@unipa.it (M.P.); elisa.costanzo01@unipa.it (E.C.); ornella.urzi@unipa.it (O.U.); vincenza.tinnirello@unipa.it (V.T.); marta.moschetti@unipa.it (M.M.); alice.conigliaro@unipa.it (A.C.); stefania.raimondo@unipa.it (S.R.); riccardo.alessandro@unipa.it (R.A.); 2 Agrumaria Corleone s.p.a., Via S. Corleone, 12—Zona Ind. Brancaccio, 90124 Palermo, Italy; vacorleone@agrumariacorleone.com

**Keywords:** lemon essential oil, lipopolysaccharide, hepatocytes, inflammation, oxidative stress, epithelial–mesenchymal transition

## Abstract

**Simple Summary:**

To date, essential oil fractions are emerging as functional compounds of interest for the food and perfume industries. The aim of this study is to evaluate the ability of citral-enriched fractions obtained from lemon essential oil (Cfr-LEO) to counteract, in healthy human hepatocytes, the activity of lipopolysaccharide (LPS), a trigger of inflammation, oxidative stress, and epithelial–mesenchymal transition. In our paper, we report that the pretreatment of hepatocytes with Cfr-LEO counteracts the effects induced by LPS. The data obtained lay the basis for the development of commercial products such as food and drink aimed at preventing or alleviating chronic conditions associated with liver dysfunction.

**Abstract:**

Lemon essential oil (LEO) is known for its aromatic and healthy properties; however, less consideration is given to the biological properties of the fractions obtained from LEO. This study aims to evaluate the ability of a citral-enriched fraction obtained from LEO (Cfr-LEO) to counteract lipopolysaccharide (LPS)-mediated inflammation, oxidative stress, and epithelial–mesenchymal transition (EMT) in healthy human hepatocytes. Human immortalized hepatocytes (THLE-2 cell line) were pretreated with Cfr-LEO and subsequently exposed to LPS at various time points. We report that the pretreatment with Cfr-LEO counteracts LPS-mediated effects by inhibiting inflammation, oxidative stress, and epithelial–mesenchymal transition in THLE-2. In particular, we found that pretreatment with Cfr-LEO reduced NF-κB activation and the subsequent proinflammatory cytokines release, ROS production, and NRF2 and p53 expression. Furthermore, the pretreatment with Cfr-LEO showed its beneficial effect in counteracting LPS-induced EMT. Taken together, these results support Cfr-LEO application in the nutraceutical research field not only for its organoleptic properties, conferred by citral enrichment, but also for its biological activity. Our study could lay the basis for the development of foods/drinks enriched with Cfr-LEO, aimed at preventing or alleviating chronic conditions associated with liver dysfunction.

## 1. Introduction

Acute liver failure and chronic liver diseases can be induced by various etiological agents and are related to the generation of inflammatory cytokines and oxygen free radicals, driving liver fibrosis [1,2,3]. A main priority for protecting the liver from injuries causing morbidity and mortality is to suppress or counteract the mechanisms that lead to the initiation and progression of fibrosis as the inflammatory and oxidative stress response and the activation of epithelial–mesenchymal transition (EMT) [3,4]. Even if the Kupffer cell-mediated activation of the hepatic stellate cells is considered the key event in the occurrence and development of liver fibrosis [3], recent evidence clearly indicates that in the injured liver the hepatocytes also can be activated to release inflammatory mediators and to undergo EMT [4]. Lipopolysaccharide (LPS), a component of the Gram-negative bacterial cell membrane, is well known to be implicated in liver injury. LPS stimulates, via Toll-like receptor 4 (TLR4) and its adapter molecules, the parenchymal and nonparenchymal liver cells to produce inflammatory mediators, thus inducing fibrogenic activity [5,6,7].

The literature data report natural compounds’ abilities to prevent or alleviate LPS-induced inflammation, oxidative stress, and EMT. Wang et al. demonstrated that the pretreatment of LPS-stimulated hepatocytes with apigenin could decrease the expression of NF-κB, TNFα, and IL-6 and increase the level of nuclear factor-kappa B inhibitor alpha IκB-α [8]. Yu Z. et al. demonstrated that daidzein, extracted from leguminous plants, exerts in LPS-induced hepatocytes anti-inflammatory properties by decreasing the expression of IL-lβ, IL-6, and TNFα, and antioxidant properties by reducing ROS and increasing superoxide dismutase (SOD) activity [9]. Chen M. et al. showed that resveratrol, a natural antioxidant polyphenol, plays an important role in the inhibition of LPS-induced EMT in mouse melanoma by downregulating NF-κB activity [10]. Ho C. et al. demonstrated the pharmacological effects of wild bitter melon (WM) on hepatic stellate cells (HSCs) activation following LPS treatment, showing that WM treatment may protect against liver fibrosis via HSC inactivation or death, thus suggesting its application for the management of liver fibrosis [11].

Interestingly, several data show that the use of natural compounds exhibits more effective biological activity when provided as a natural mixture rather than as a single constituent [12]. Among the natural mixtures, the essential oils (EOs) are described as highly versatile. EOs are used as fragrances in cosmetic products as well as active ingredients in a healthy diet for their multiple biological properties (antibacterial, antiviral, anti-inflammatory, and antioxidant). Their multi-target activity has stimulated over the years their application in both the prevention and therapy of several diseases [12]. Although the functional activities of whole EOs obtained from different plants have been described in several cellular models [13,14], to the best of our knowledge, the current literature regarding essential oil fractions is mainly focused on isolation techniques and description of the molecular profile, but still lacks functional studies. The protective effect of lemon EO in counteracting inflammation and oxidative stress has been already reported [15,16]. Among the biologically active compounds found in lemon EO, citral, a well-known and well-characterized mixture of the two aldehydes geranial and neral, is emerging as a key compound in the lemon EO molecular profile due to its contribution to the lemon flavor and its biological properties [17,18,19,20,21]. In our recently published study, we revealed the beneficial properties of four selected and mixed citral-enriched fractions of lemon essential oil (Cfr-LEO), showing their ability to reduce the expression of pro-inflammatory mediators and to decrease oxidative stress in LPS-stimulated macrophages [22].

The present study was designed to explore whether the Cfr-LEO exhibits a liver-protective role and to elucidate the potential underlying molecular mechanisms.

We hypothesized that Cfr-LEO protects human hepatocytes from LPS-induced inflammation, oxidative stress injury, and EMT, all events strictly related to each other and correlated with liver dysfunction. Cfr-LEO’s capacity to safeguard hepatocytes from hepatotoxic triggers makes it a relevant product in preventing liver damage.

## 2. Materials and Methods

### 2.1. Cell Culture

**Figure 1:**
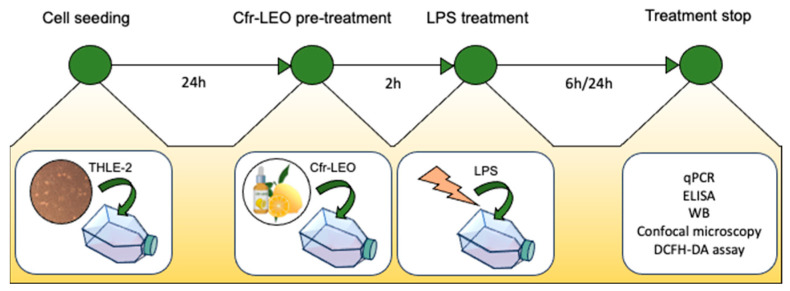
Graphical representation of the experimental design.

THLE-2 (ATCC CRL-2706™, LGC Standards) human cells isolated from the left lobe of a healthy liver and immortalized with the catalytic subunit of human telomerase hTERT were maintained in RPMI 1640 medium (Euroclone, UK) supplemented with 10% fetal bovine serum (FBS, Euroclone, UK), 1% penicillin (100 U/mL) and streptomycin (100 µg/mL), 0.3 mL human recombinant epidermal growth factor (hEGF) (10 µg/mL), and 0.4 mL phosphoethanolamine (PEA) (100 µg/mL). The cell line was tested for Mycoplasma using the Hoechst staining and the N-GARDE Mycoplasma PCR reagent set (Euroclone), it was authenticated with a morphology check and cell proliferation rate evaluation, and bacteria contamination was excluded. Cells were grown on a coating made of 0.03 mg/mL bovine collagen type I (Advanced Biomatrix, San Diego region, CA, USA) and 0.01 mg/mL bovine serum albumin (Sigma-Aldrich, St Louis, MO, USA). Cfr-LEO was obtained as previously described [22] and THLE-2 cells were consequently treated, as described in the graphical representation shown in Figure 1.

### 2.2. MTT (3-[4,5-Dimethylthiazol-2-yl]-2,5 Diphenyl Tetrazolium Bromide) Assay

The MTT assay is used to measure cellular metabolic activity. Accordingly, MTT was performed to select the Cfr-LEO and LPS doses to use for treating the hepatocytes. THLE-2 were seeded in triplicate at 3 × 10^4^ cells per well in 24-well plates; after 24 h and 48 h of seeding, cells were treated for further 24 and 48 h with different concentrations of Cfr-LEO (0.005%, 0.01%, 0.02%, 0.05%) prepared as described in the following. Cfr-LEO 100% was first solubilized in a solution of FBS 95% and DMSO 5% to bring it to a final concentration of 1%, then further diluted in cell culture medium to obtain the following final concentrations: 0.005%, 0.01%, 0.02%, and 0.05%. In the MTT assay, untreated cells and cells treated with the highest used concentration of DMSO (0.25%) were used as controls. The MTT solution was prepared as a 5 mg/mL stock solution in phosphate-buffered saline (pH 7.4) and filtered (0.22 μm, Millipore, Burlington, MA, USA). At the end of the treatment, the warmed (37 °C) MTT stock solution was added to each well according to the manufacturer’s instruction. The plates were then incubated at 37 °C for 3 h and stopped with a solution of 0.4% HCl in isopropanol. The absorbance was measured with an ELISA reader at 540 nm (Microplate Reader, BioTek, Winooski, VT, USA). MTT assay was then performed to select LPS concentration (100 ng/mL, 250 ng/mL, 500 ng/mL, and 1000 ng/mL); 24 h after seeding, cells were treated with LPS for 24 h. After the treatment, the MTT stock solution was warmed at 37 °C and added to the cell according to the manufacturer’s instruction. The plates were then incubated at 37 °C for 3 h and stopped with a solution of 0.4% HCl in isopropanol. The absorbance was measured with an ELISA reader at 540 nm (Microplate Reader, BioTek, Winooski, VT, USA). Values are expressed as a percentage of cell growth versus control (untreated cells).

### 2.3. RNA Isolation and Quantitative Real-Time PCR

Levels of interleukin-1β (IL-1β), interleukin (IL-6), and tumor necrosis factor (TNFα) were measured using quantitative real-time PCR (qRT-PCR). THLE-2 were seeded at 8 × 10^4^ per well in 12-well plates and, after 24 h, treated with Cfr-LEO 0.01% following this protocol: pretreatment for 2 h with Cfr-LEO (0.01% and 0.02%) before their exposure to LPS (100 ng/mL) for 6 h, without oil removal. After the treatment, total RNA was isolated using the illustra TM RNAspin mini-RNA isolation kit (GE Healthcare, Little Chalfont, Buckinghamshire, UK). Then, total RNA was reversely transcribed into cDNAs with a high-capacity cDNA reverse transcription kit (Applied Biosystems, Foster City, CA, USA). The cDNA was analyzed by performing qRT-PCR with SYBR Green Master mix (Applied Biosystems, Foster City, CA, USA) and the primers reported in Table 1. The cycling qualifications were as follows: 95 °C for 30 s, then 40 cycles of 95 °C for 5 s, then 55 °C for 30 s, and then 72 °C for 1 min. All reactions were performed in duplicate, and at least three independent experiments were analyzed. Quantitative analysis was performed calculating the expression of IL-1β, IL-6, and TNFα relative to the endogenous housekeeping gene GAPDH. Relative quantification was calculated using the comparative threshold cycle method (ΔΔCT).

### 2.4. ELISA (Enzyme Immunosorbent Assay)

The amount of IL6 released in the conditioned medium of THLE-2 cells was quantified using the specific ELISA kit (Thermo Fisher Scientific, Waltham, MA USA). THLE-2 cells were seeded at 8 × 10^4^ per well in 12-well plates, and after 24 h, cells were pretreated for 2 h with Cfr-LEO (0.01%) before their exposure to LPS (100 ng/mL) for 6 h. The LPS treatment was carried out without Cfr-LEO removal, and, at the end of the experimental time, the conditioned medium was collected and centrifuged to remove cellular debris at 300 g for 5 min. The ELISA assay was performed according to the manufacturer’s instructions. The absorbance reading was executed with the spectrophotometer, using 450 nm as a wavelength.

### 2.5. Western Blot

THLE-2 were seeded at 3 × 10^5^ cells on a coating of type I bovine collagen in the culture flasks (T25). Cells were pretreated for 2 h with Cfr-LEO (0.01% or 0.02%) and then with LPS (100 ng/mL or 1000 ng/mL) for 30 min, 6 h, and 24 h, depending on the experimental downstream investigations; THLE-2 were recovered from the culture flask using a cell scraper. Protein lysate was obtained by adding lysis buffer (Tris-HCl pH 7.6, 50 mM, NaCl 300 mM, TritonX-100 0.5%, PMSF 1X, leupeptin 1X, aprotinin 1X, phosphatase inhibitors 1X (phosphatase inhibitor cocktail 10X), and H2O milliQ). Protein quantification was carried out using Bradford assay, and the reading was executed on the biophotometer at a wavelength of 595 nm. H_2_O, sample buffer (4X), and reducing agent (10X) were added to the quantized proteins, which were consequently loaded onto SDS-page Bolt TM 4–12% Bis-Tris Plus (Invitrogen). Proteins were transferred on a nitrocellulose membrane (MCE, Invitrogen, Life Technologies, Rockford, IL, USA) in the presence of the transfer buffer (10X Tris-glycine, 20% methanol, H_2_O milliQ). The membrane was incubated in BSA blocking solution for 90 min and probed overnight with primary antibodies: anti-NRF2, anti-P53, anti-NF-κB p65 (Novus Biologicals, E. Briarwood Avenue Centennial, CO, USA), anti-p-NF-*κB* p65 (Invitrogen, Thermo Fisher Scientific, Waltham, MA, USA), anti-vimentin, anti-N-cadherin (Cell Signalling Technology, Lane Danvers, MA, USA), and anti-β-actin and anti-GAPDH (Santa Cruz Biotechnology, Dallas, TX, USA). The membrane was incubated with HRP-conjugated goat anti-rabbit or anti-mouse secondary antibodies (1:1000 dilution; Thermo Fisher Scientific, Waltham, MA, USA). Chemiluminescence was detected using the Amersham ECL Western Blotting Detection Reagent (GE Healthcare Life Sciences, Little Chalfont, Buckinghamshire, UK) chemiluminescence solution. The signal detection was performed with the “ChemiDocTMMP” (Imaging System) and the densitometric analysis was carried out with the “Image J” 1.48v software.

### 2.6. Confocal Microscopy

THLE-2 were grown and seeded at 1 × 10^5^ cells into chambers of 4.2 cm^2^ (Thermo Fisher Scientific, 155380), and after 24 h, cells were pretreated with Cfr-LEO for 2 h and then treated with 100 ng/mL of LPS for 30 min. At the end of the treatment, THLE-2 were fixed with PFA 4% for 10 min and then washed three times with PBS. Furthermore, THLE-2 were permeabilized with triton 0.1% for 2 min, and then they were incubated with BSA 1% for 30 min. After that, THLE-2 cells were labeled with anti-NF-κB p65 (Novus Biologicals) (dilution 1:100) for 1 h and then with the fluorescent secondary antibody Alexa fluor 594 (Life Technologies, Thermo Fisher Scientific) (dilution 1:500) for 1 h. Afterward, the nuclei were labeled with Hoechst 33342, trihydrochloride, trihydrate (Invitrogen, Thermo Fisher Scientific) (dilution 1:1000), and the cytoskeleton was labeled with Actin Green 488 Ready Probes reagent (Invitrogen, Thermo Fisher Scientific) for 30 min. At the end of the labeling method, cells were observed with confocal microscopy (Nikon A1).

### 2.7. Dichlorodihydrofluorescein Diacetate (DCFH-DA) Assay

Cell-permeable 2′,7′-dichlorodihydrofluorescein diacetate (H2DCFDA) was used to detect reactive oxygen species (ROS) in cells. THLE-2 were seeded 3 × 10^4^ per well in a 24-well plate. After 24 h, cells were pretreated for 2 h with Cfr-LEO (0.01%) and for 6 h with LPS (100 ng/mL). At the end of the treatment, the cell culture medium was removed, and DCFH-DA was added at a final concentration of 10 μM in the cell culture medium FBS-depleted. THLE-2 cells were incubated with DCFH-DA at 37 °C at 5% of CO_2_ in the dark for 30 min and then were washed with phosphate-buffered saline (PBS) three times to completely remove excess DCFH-DA. ROS production was observed using fluorescence microscopy (Nikon Eclipse Ti) and quantified using ImageJ software.

### 2.8. Statistical Analysis

Data are reported as means ± SD. Comparisons were made by performing the one-way ANOVA multiple comparisons test with the Holm–Šídák method or the two-way ANOVA (or mixed model) multiple comparisons test with the Tukey method. Analyses were conducted using GraphPad Prism Version 10.1.0 (316). Values were considered statistically significant when *p* ≤ 0.05.

## 3. Results

### 3.1. In Vitro Anti-Inflammatory Effects of Cfr-LEO

**Figure 2:**
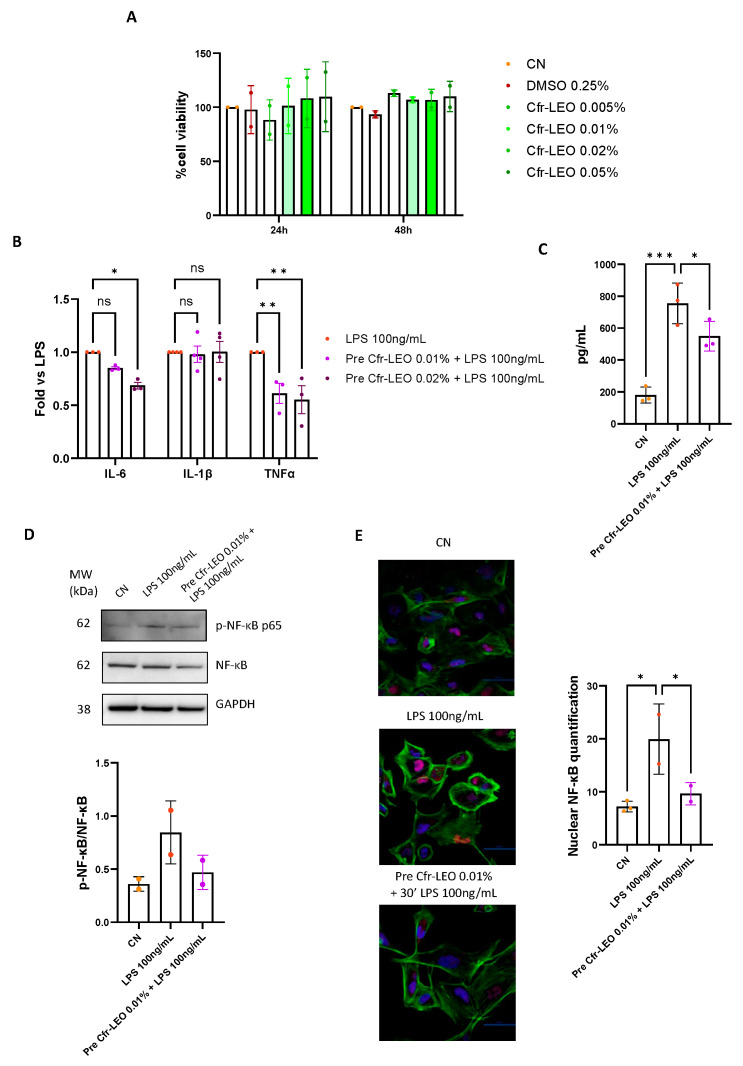
(**A**) Hepatocyte cell viability after exposure to Cfr-LEO. THLE-2 viability was measured with MTT assay after 24 and 48 h of treatment with different concentrations of Cfr-LEO (0.005%, 0.01%, 0.02%, and 0.05%). The values were plotted as the percentage of cell viability versus untreated cells (CN). Values are the mean ± SD of two biological replicates. (**B**,**C**) Anti-inflammatory effects of Cfr-LEO on LPS-stimulated THLE-2. (**B**) The effect of Cfr-LEO on inflammatory cytokines expression was assessed with qRT-PCR analysis. THLE-2 cells were treated for 2 h with 0.01% and 0.02% Cfr-LEO and then exposed to LPS 100 ng/mL for 6 h. Values are reported as fold change versus cells treated with LPS alone and are the mean ± SD of three biological replicates. (**C**) IL-6 protein level was measured with ELISA in the conditioned medium of THLE-2 cells treated for 2 h with 0.01% Cfr-LEO and then exposed to LPS 100 ng/mL for 6 h. Values are plotted as fold change versus cells treated with LPS alone (LPS). Values are the mean ± SD of three biological replicates. The statistical significance was calculated versus the LPS-treated cells (LPS) by using Student’s *t*-test. (**D**,**E**) Effects of the Cfr-LEO pretreatment on the LPS-induced NF-κB activation. (**D**) Western blot showing the level of phosphorylated p65 subunit of NF-κB (p-NF-κB p65) and total p65 subunit of NF-κB (NF-κB p65) in THLE-2 cells treated for 30 min with 100 ng/mL LPS alone (LPS 100 ng/mL) or pretreated for 2 h with 0.01% Cfr-LEO (pre Cfr-LEO 0.01% + LPS 100 ng/mL). GAPDH was used as the loading control. CN: untreated cells used as control. The values reported in the densitometric analysis are the mean (±SD) of the analyzed protein normalized vs. loading control from at least two independent experiments. (**E**) Representative micrographs from confocal fluorescence microscopy of THLE-2 cells treated for 30 min with 100 ng/mL LPS alone (LPS 100 ng/mL) or pretreated for 2 h with 0.01% Cfr-LEO (pre Cfr-LEO 0.01% + LPS 100 ng/mL). THLE-2 cells were stained for NF-κB (red) and were labeled with Hoechst to observe the nucleus (blue) and with Actin Green for the cytoskeleton (green). CN: untreated cells used as control. The values reported in the densitometric analysis are the mean (±SD) of the analyzed micrographs from at least two observations. Colored dots represent the number of replicates for each condition. ns = not significant, * *p* ≤ 0.05, ** *p* ≤ 0.01, and *** *p* ≤ 0.001.

The effects of the Cfr-LEO treatment on THLE-2 cell viability were preliminarily evaluated by performing an MTT assay. The results, reported in Figure 2A, show that cell treatment with 0.005%, 0.01%, 0.02%, and 0.05% Cfr-LEO for both 24 h and 48 h did not affect THLE-2 cell viability; thus, according to our previously published data [22], the Cfr-LEO concentrations ranging from 0.01% to 0.02% were employed for subsequent experiments. Similarly, after performing MTT assay on THLE-2 treated with LPS for 24 h (Supplementary Figure S1), the doses of 100 ng/mL and 1000 ng/mL, in accordance with our previous data reported in the literature [22], were used in the present study.

To determine whether Cfr-LEO exhibited protective effects against LPS-induced inflammatory response in hepatocytes, we first demonstrated that the treatment of THLE-2 cell lines with LPS 100 ng/mL significantly upregulated the expression of the inflammatory mediators IL-6, IL-1β, and TNFα, whereas the treatment with Cfr-LEO 0.01% and 0.02% did not affect the expression of the above-mentioned cytokines, as shown in Supplementary Figure S2. Subsequently, we analyzed the effects of Cfr-LEO in counteracting LPS-induced upregulation of IL-6, IL-1β, and TNFα. As shown in Figure 2B, we found that the pretreatment of THLE-2 cells for 2 h with Cfr-LEO significantly inhibited the expression TNFα induced by 6 h LPS treatment, while no effects were observed for IL-1β. A possible explanation for the different effects exerted by Cfr-LEO on IL-1β expression could be linked to a different LPS-mediated induction due to a different basal expression of the cytokine. Moreover, alternative pathways responsible for IL-1β transcription [23] could affect the cytokine expression.

Concerning IL-6, we found a significant decrease in the cytokine expression in the Cfr-LEO 0.02% pretreated condition when compared with LPS-treated cells and a not significative decrease trend for IL-6 expression in THLE-2 cells pretreated for 2 h with Cfr-LEO 0.01%. Despite this, we found, by performing ELISA assay, a significative decrease in IL-6 release in the Cfr-LEO 0.01% pretreated condition when compared with LPS-treated cells (Figure 2C). The TNFα expression at the protein level was not detectable in any analyzed condition. Overall, these results suggest that Cfr-LEO exerts an anti-inflammatory effect in LPS-stimulated hepatocytes, suggesting its hepatoprotective role.

Furthermore, to understand the molecular mechanisms responsible for the anti-inflammatory effects mediated by Cfr-LEO, we investigated the consequences of the Cfr-LEO pretreatment on the LPS-induced activation of nuclear factor κB (NF-κB), a well-known target of the LPS/TLR4 signaling pathway [24]. As shown in Figure 2D, the pretreatment of THLE-2 for 2 h with 0.01% Cfr-LEO inhibited the phosphorylation of NF-κB induced by the LPS treatment. To confirm this data, confocal analysis was performed and revealed that pretreatment of THLE-2 for 2 h with 0.01% Cfr-LEO clearly inhibited the nuclear translocation and the consequent activation of NF-κB when compared with LPS-treated cells (Figure 2E).

### 3.2. Antioxidant Effects of Cfr-LEO

**Figure 3:**
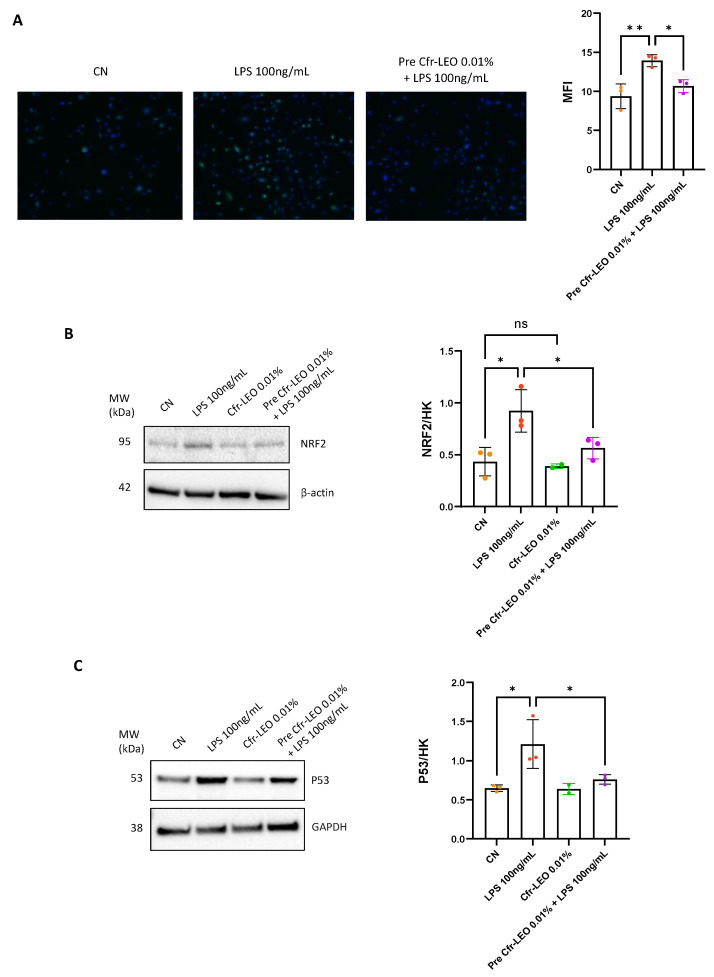
Antioxidant effects of Cfr-LEO on LPS-stimulated THLE-2. (**A**) The antioxidant effects of Cfr-LEO were evaluated with a DCFDA—Cellular ROS assay kit probe. The hepatocytes were treated for 6 h with 100 ng/mL LPS alone (LPS) or pretreated for 2 h with 0.01% Cfr-LEO (pre Cfr-LEO 0.01% + LPS 100 ng/mL). The intensity of the green fluorescence is proportional to the amount of ROS present in the sample. Values of mean fluorescence intensity reported in the histogram were obtained by analyzing images with the Image J software and are the mean ± SD of three independent experiments. Values are plotted as fold change versus cells treated with LPS alone (LPS 100 ng/mL). Nuclei were labeled with Hoechst (blue). (**B**,**C**) Western blot analysis of NRF2 and p53 treated for 6 h with 100 ng/mL LPS alone (LPS 100 ng/mL) or pretreated for 2 h with 0.01% Cfr-LEO (pre Cfr-LEO 0.01% + LPS 100 ng/mL). GAPDH was used as the loading control. The values reported in the densitometric analysis are the mean (±SD) of the analyzed protein normalized vs. loading control from at least three independent experiments. CN: untreated cells used as control. Colored dots represent the number of replicates for each condition. ns = not significant * *p* ≤ 0.05 and ** *p* ≤ 0.01.

Since it is known that the LPS-induced TLR4/NF-κB signaling pathway also promotes the production of reactive oxygen species [25], we investigated whether Cfr-LEO could exhibit protective effects against LPS-induced oxidative stress. As shown in Figure 3A, we observed that the pretreatment of THLE-2 for 2 h with 0.01% Cfr-LEO inhibited the production of intracellular ROS compared with the LPS-treated cells. Interestingly, we found that the Cfr-LEO-pretreated hepatocytes did not respond to the pro-oxidant state promoted by LPS, as demonstrated by the expression levels of NRF2 and p53, which appeared comparable to control cells (Figure 3B,C).

### 3.3. Cfr-LEO Protects Hepatocytes from the LPS-Induced EMT

**Figure 4:**
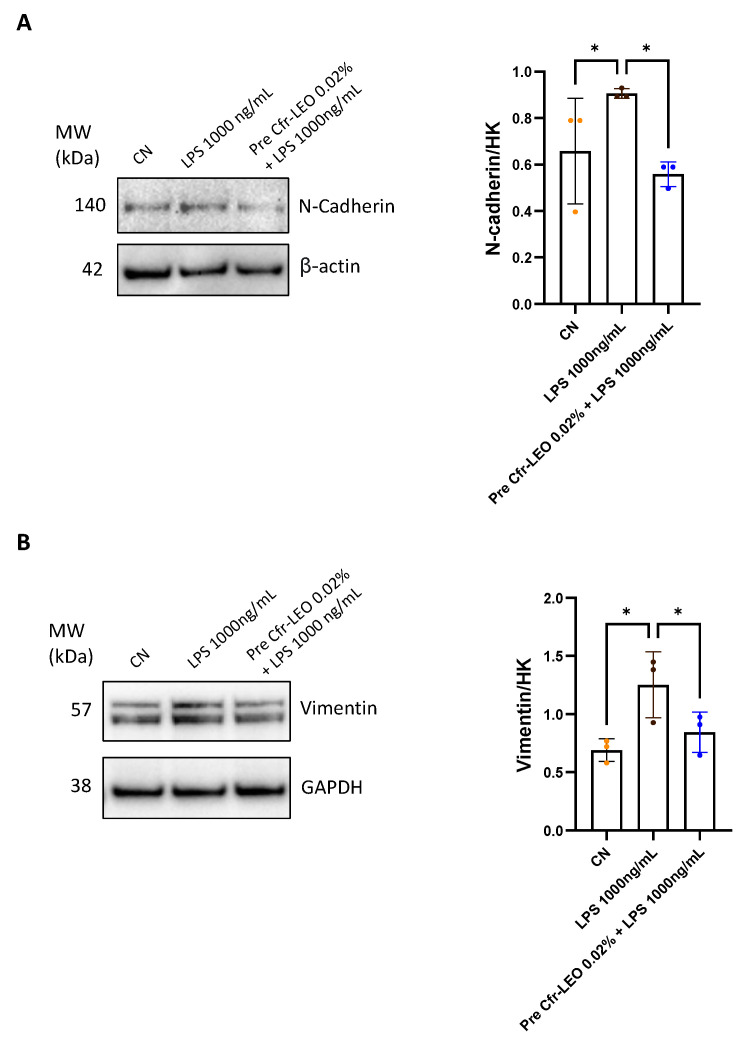
Analysis of the EMT inhibition properties of Cfr-LEO. (**A**,**B**) Western blot analysis of N-cadherin and vimentin in THLE-2 pretreated with Cfr-LEO (0.02%) and treated with LPS (1000 ng/mL) for 24 h. β-actin and GAPDH were used as the loading controls. The values reported in the densitometric analysis are the mean (±SD) of the analyzed protein normalized vs. loading control from at least three independent experiments. CN: untreated cells used as control. Colored dots represent the number of replicates for each condition. * *p* ≤ 0.05.

Several studies have demonstrated that in the development and progression of chronic liver diseases, inflammation and fibrosis are often concomitant [26]. Even if most of the available data indicate that the activated hepatic stellate cells are the key players in the fibrogenic process, some evidence suggests that hepatocytes may also acquire a pro-fibrotic behavior through EMT, indicating this process as a potential target to develop new strategies to prevent liver fibrosis [27,28]. Data in the literature report that the LPS induces EMT in epithelial target cells [11,29,30]. To evaluate the ability of Cfr-LEO to protect hepatocytes from the LPS-induced EMT, THLE-2 were treated for 2 h with 0.02% of Cfr-LEO and then for 24 h with 1000 ng/mL of LPS, a dose selected based on data reported in the literature [11,29,30]. The data reported in Figure 4A,B show that the modulation of N-cadherin and vimentin induced by LPS was significantly counteracted by the pretreatment of THLE-2 with Cfr-LEO.

## 4. Discussion

To date, nutraceuticals are expected to play a central role in preventive healthcare, representing an exciting new opportunity to converge food and pharma. The interest of the nutraceutical industry is focused on discovering natural compounds that possess both biological and organoleptic properties, thus introducing “smart food” that can be included in a health-promoting diet. Medicinal plants and their derivatives such as essential oils contain a wide variety of bioactive compounds that possess beneficial properties such as antioxidant and anti-inflammatory activities [31,32]. It is known that several essential oils obtained from different plants have hepatoprotective effects contrasting the pro-inflammatory, pro-oxidant, and pro-fibrotic activities of hepatotoxic molecules [13,14].

Recently, Pucci et al. reported the biological properties of Cfr-LEO on LPS-activated macrophages, thus highlighting its application not only in healthcare for its beneficial properties but also in the nutraceutical industry, as a natural food additive for its organoleptic properties, conferred by citral enrichment.

In this study, we further confirmed the biological properties of Cfr-LEO on a model of healthy human hepatocytes. We demonstrated, first, that Cfr-LEO counteracts the expression of pro-inflammatory cytokines such as IL-6 and TNFα in LPS-stimulated hepatocytes by preventing the LPS-induced NF-κB phosphorylation and nuclear translocation.

We hypothesized that these effects could be due to Cfr-LEO’s ability to inhibit the TLR4/NF-κB pathway. Indeed, to date, several molecules from plants and herbs of traditional Chinese medicine, such as berberine, atractylenolide I, and zhankuic acid A, have been described as TLR4 antagonist molecules. Moreover, curcumin from Curcuma longa, sulforaphane and iberin from cruciferous vegetables, xanthohumol from hops and beer, and celastrol from Tripterygium wilfordii have already also been identified as TLR4 antagonists [33]. Our results suggest that Cfr-LEO, similarly to other natural compounds, could act as a TLR4 antagonist, thus hindering LPS binding to the receptor and therefore counteracting the downstream targets of LPS/TLR4 signaling, among which are NF-κB and its target genes involved in the inflammatory response. These data lay the basis for further studies aimed at demonstrating the role of Cfr-LEO in preventing TLR4-associated inflammatory response. Moreover, we demonstrated Cfr-LEO’s protective effect on hepatocytes’ oxidative stress. Oxidative stress is at the basis of the establishment of lipid peroxidation and the accumulation of lipid droplets, the main causes of the development of liver diseases such as metabolic syndromes and NAFLD, fibrosis, and HCC [34].

We found that pretreatment with Cfr-LEO counteracts LPS-induced oxidative stress by reducing the ROS level. Moreover, we found that NRF2 and p53, factors involved in the oxidative stress defense, showed comparable expression levels in both control and Cfr-LEO-pretreated cells. Taken together, these results support the hypothesis that Cfr-LEO could act as a TLR4 antagonist by an upstream blocking of the TLR4 signaling cascade, thus making cells unresponsive to LPS-induced oxidative stress. Further studies will be necessary to confirm our preliminary hypothesis.

Lastly, we demonstrated that Cfr-LEO counteracts the expression of EMT markers in LPS-stimulated hepatocytes [35], thus highlighting the ability of Cfr-LEO to prevent the establishment of a fibrotic environment in the liver. Liver fibrosis and cirrhosis are the ultimate consequences of chronic hepatic injury induced by various etiological agents, and they are associated with significant morbidity and mortality in the world [36,37]. Liver fibrosis is characterized by abnormally enhanced tissue deposition of extracellular matrix (ECM) components. Oxidative stress, inflammation, and EMT play a key role in fibrogenesis induction [28,38,39]. Although liver fibrosis has been reported to be reversible at early stages, it becomes irreversible with advanced disease, leading to the malignant transformation of cells toward a “cancer phenotype” [29,40]. To date, the mechanism of liver fibrosis has been extensively studied, and in recent years, many strategies have emerged as crucial to inhibit the occurrence and development of liver fibrosis, including inhibition of hepatic stellate cells (HSCs) activation and proliferation, reduction in ECM overproduction, and acceleration of ECM degradation. However, to date, effective antifibrotic therapies are lacking [41]. In the current study, we found that the mesenchymal markers vimentin and N-cadherin show comparable expression levels in both controls and Cfr-LEO-pretreated cells, thus further supporting the ability of Cfr-LEO to protect cells from hepatotoxic LPS stimuli.

Our future studies will be focused on evaluating the beneficial effects of selected Cfr-LEO components to understand which of them could act as a “TLR4 antagonist,” thus determining the observed protective effects of the fraction. Therefore, having evaluated its nontoxicity and beneficial properties in a healthy human hepatocytes cellular model, we confirm that Cfr-LEO can certainly be applied not only in the agri-food industry for its organoleptic properties, but it can also represent a preventive tool for improving human health, exerting a protective effect against hepatotoxic stimuli [42,43], suggesting its possible beneficial effect for a complex organ frequently exposed to damage, such as the liver.

In conclusion, this work lays the foundation for the development of specific foods/drinks, made from scientifically tested citrus essential oils, aimed at preventing or alleviating chronic inflammatory conditions associated with liver dysfunction. The product could be a dried spray that, added to food products, can give the same healthy properties. The future identification of the specific compounds of Cfr-LEO responsible for the health effects observed could lead also to more targeted food/drink formulations with beneficial properties.

## Figures and Tables

**Table 1:** Oligonucleotides used in qRT-PCR (9); 60° is the temperature of the annealing.

Primers.	Forward	Reverse
GAPDH	ATGGGGAAGGTGAAGGTCG	GGGTCATTGATGGCAACAATAT
IL-6	GGTACATCCTCGACGGCATCT	GTGCCTCTTTGCTGCTTTCAC
IL-1β	ACAGATGAAGTGCTCCTTCCA	GTCGGAGATTCGTAGCTGGAT
TNFα	CCAGGCAGTCAGATCATCTTCTCTC	AGCTGGTTATCTCTCAGCTCCAC

## Data Availability

Data are contained within the article and Supplementary Materials. Data sharing not applicable.

## Supplementary Materials

The following supporting information can be downloaded at: https://www.mdpi.com/article/10.3390/biology12121535/s1, Figure S1: MTT assay after 24 h of treatment with different concentrations of LPS (100 ng/mL, 250 ng/mL, 500 ng/mL, and 1000 ng/mL); Figure S2: qRT-PCR analyses of THLE-2 cells treated for 6 h LPS 100 ng/mL and for 8 h with 0.01% and 0.02% Cfr-LEO.
