# Supplementary material for: Citral-Enriched Fraction of Lemon Essential Oil Mitigates LPS-Induced Hepatocyte Injuries
Source: Biology (Basel). 2023 Dec 17;12(12):1535. doi: 10.3390/biology12121535 (PMC10740427; doi:10.3390/biology12121535)
Supplement: Supplementary file 1 [file biology-12-01535-s001.zip › biology-2721207-supplementary.pdf]

Supplementary Figures

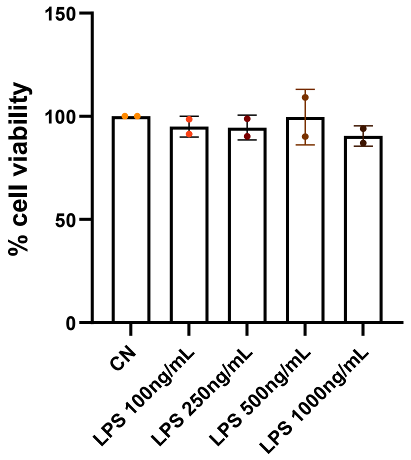


*Figure S1. Hepatocyte cell viability after exposure to LPS. THLE-2 viability was measured by MTT assay after 24 of treatment with different concentrations of LPS (100ng/mL, 250ng/mL, 500ng/mL, 1000ng/mL). The values were plotted as the percentage of cell viability versus untreated cells (CN). Values are the mean ± SD of two biological replicates.*

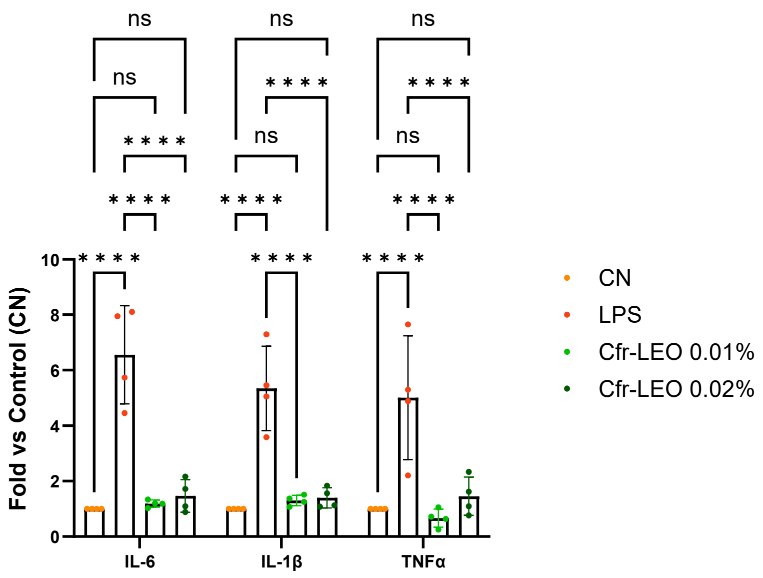


*Figure S2. The effect of LPS 100ng/mL, Cfr-LEO 0.01%, and Cfr-LEO 0.02% on inflammatory cytokines expression was assessed by qRT-PCR analyses. THLE-2 cells were treated for 6h LPS 100ng/mL and for 8h with 0.01% and 0.02% Cfr-LEO. Values are reported as fold change versus cells treated with LPS alone and are the mean ± SD of three biological replicates.*
